# SHP2 inhibitor PHPS1 protects against atherosclerosis by inhibiting smooth muscle cell proliferation

**DOI:** 10.1186/s12872-018-0816-2

**Published:** 2018-04-27

**Authors:** Jia Chen, Zhiyong Cao, Jingshu Guan

**Affiliations:** 1 Department of Cardiology, Shanghai Baoshan Hospital of Integrated Traditional Chinese and Western Medicine, Friendship Road 181, Baoshan District, Shanghai, China; 2 Department of Cardiology, Shanghai Navy 411 Hospital, Shanghai, China

**Keywords:** SHP2, PHPS1, Atherosclerosis, Smooth muscle cells, Proliferation, ERK

## Abstract

**Background:**

Smooth muscle cells play an important role in the development of atherosclerosis. SHP2 is known to regulate the proliferation and migration of smooth muscle cells. The purpose of this study was to determine whether the SHP2 inhibitor PHPS1 has a pro-atherosclerotic or an atheroprotective effect in vivo and in vitro.

**Methods:**

After exposure to a high-cholesterol diet for 4 weeks, LDL receptor-deficient (Ldlr^−/−^) mice were exposed to the SHP2 inhibitor PHPS1 or vehicle. Body weight, serum glucose and lipid levels were determined. The size and composition of atherosclerotic plaques were measured by en face analysis, Movat staining and immunohistochemistry. The phosphorylation of SHP2 and related signaling molecules was analyzed by Western blot. Mechanistic analyses were performed in oxLDL-stimulated cultured vascular smooth muscle cells (VSMCs) with or without 10 mM PHPS1 pretreatment. Protein phosphorylation levels were detected by Western blot, and VSMC proliferation was assessed by BrdU staining.

**Results:**

PHPS1 decreased the number of atherosclerotic plaques without significantly affecting body weight, serum glucose levels or lipid metabolism. Plaque composition analysis showed a significant decrease in the number of VSMCs in atherosclerotic lesions of Ldlr^−/−^ mice treated with PHPS1. Stimulation with oxLDL induced a dose-dependent increase in the number of VSMCs and in SHP2 and ERK phosphorylation levels, and these effects were blocked by PHPS1.

**Conclusion:**

The SHP2 inhibitor PHPS1 exerts a protective effect against atherosclerosis by reducing VSMC proliferation via SHP2/ERK pathway activation.

**Electronic supplementary material:**

The online version of this article (10.1186/s12872-018-0816-2) contains supplementary material, which is available to authorized users.

## Background

Cardiovascular disease is a threat to human health and seriously impacts quality of life [1]. Atherosclerosis (AS) is a major pathological basis of cardiovascular and cerebrovascular diseases [2]. Although the mechanism underlying AS is complex, the basic pathology of AS is fibrous proliferation caused by the presence of progressive lipid deposits, accumulation of inflammatory cells and deposition of extracellular matrix [3, 4]. AS is not a separate pathological process; rather, it is regulated by a variety of cells and intracellular signaling pathways [5]. Smooth muscle cells (SMCs) play an important role in the development of AS [6, 7].

Protein tyrosine phosphorylation and dephosphorylation play significant roles in numerous intracellular signaling pathways and are influenced by several opposing kinases, such as protein tyrosine kinases (PTKs) and protein tyrosine phosphatases (PTPs) [8, 9]. PTKs catalyze tyrosine phosphorylation, whereas PTPs catalyze dephosphorylation [10]. Src homology 2 domain-containing protein tyrosine phosphatase 2 (SHP2), also known as protein tyrosine phosphatase N 11 (PTPN11), is an important PTP that acts downstream of various growth factors, cytokines and tyrosine kinases. It plays an integral role in multiple cellular events that regulate various functions, including migration, differentiation, survival and metabolism [11–13]. Schramm found that pharmacological reduction of SHP2/FAK/Akt/mTOR signaling at all levels of the signaling cascade effectively prevents cardiomyocyte hypertrophy [14]. In recent years, SHP2 inhibitors have become available for research applications. A SHP2 inhibitor phenylhydrazono pyrazolone sulfonate 1 (PHPS1) is a potent and cell permeable inhibitor that is specific for SHP2 over the closely related tyrosine phosphatases SHP1 and PTP1B [15]. In neutrophils, inflammatory monocytes and pDCs, the level of SHP2 expression is much lower than SHP1 [16]. PTP1B is an enzyme that negatively regulates insulin signaling and is likely involved in the pathways leading to insulin resistance [17]. PHPS1 remarkably suppresses E2-induced gene transcription, rapid DNA synthesis and late effects on cell growth. The finding introduces a new mechanism for SHP2 oncogenic action and sheds new light on extranuclear ER-initiated actions in breast cancer [18]. SHP2 regulates the acute pulmonary inflammation induced by cigarette smoke through the ERK1/2 pathway. PHPS1 significantly inhibits ERK1/2 activation and attenuates the inflammatory response induced in mouse lungs [19].

The purpose of this study was to determine whether the SHP2 inhibitor PHPS1 has a pro-atherosclerotic or an atheroprotective effect in vivo and in vitro by evaluating the effect of phosphorylation or dephosphorylation on the development of AS in LDL receptor-deficient (Ldlr^−/−^) mice.

## Methods

### Animal preparation

Ldlr^−/−^ (005061) mice were purchased from Jackson Laboratory. All animal protocols were approved by the Ethics Committee for Animal Experimentation at the Second Military Medical University. Five mice were housed in a cage on a 12-h light/dark cycle with free access to water and food. The animals received a high-fat diet containing 1.25% cholesterol for 4 weeks. The SHP2 inhibitor PHPS1 was purchased from Sigma-Aldrich. PHPS1 was dissolved in saline with 0.5% DMSO. Thirty mice were randomly divided into three groups: an AS group, an AS+Vehicle group and an AS+PHPS1 group. Mice in the AS+PHPS1 group received an intraperitoneal (i.p.) injection of 3 mg/kg PHPS1 every day during the last week on the high-fat diet, and those in the AS+Vehicle group received an equal volume of saline with 0.5% DMSO on the same days.

### Measurement of serum glucose and lipids

The mice were euthanized with an overdose of pentobarbital. Blood samples were collected in EP tubes containing 2 mM EDTA and centrifuged (13,000 *g*) for 15 min at 4 °C. Plasma aliquots were separated and stored at − 80 °C. The serum glucose level was determined using the glucose oxidase method (Beckman). Levels of triglycerides (TG), total cholesterol (TC), low-density lipoprotein cholesterol (LDL-C) and high-density lipoprotein cholesterol (HDL-C) were determined by high-performance liquid chromatography (HPLC).

### AS quantification

Quantification of AS in the heart and aorta was performed by staining. Briefly, the heart and aorta were perfused with phosphate-buffered saline (PBS) from the left ventricle and dissected from the aortic arch to the iliac bifurcation. The adventitia was carefully cleaned from the surrounding material. The aorta was sliced into sections, fixed in 4% paraformaldehyde for 48 h, stained with Oil Red O, incubated for 30 min at 37 °C, and washed with PBS several times; the area of the atherosclerotic lesion was compared to the total area of the aorta using a dissection microscope and Adobe Photoshop software.

The heart was embedded in optimal cutting temperature (OCT) compound and frozen at − 80 °C. Serial sections (8 μm) were taken from the aortic sinus and valve region, and sections in which all three valve leaflets were visible were used for Movat staining.

### Western blot analysis

Protein samples (30 μg) from cultured cells or aortic specimens were electrophoresed on a 12% polyacrylamide gel by SDS-PAGE and blotted onto a nitrocellulose membrane. After being blocked with 5% BSA for 2 h at room temperature, the membrane was incubated overnight at 4 °C with primary antibodies (PRS3901, M5670, SAB4500491, and SAB4502398; 1:1,000 dilution; Sigma-Aldrich), washed with TBST 3 times, incubated with anti-rabbit secondary antibody (1:5,000 dilution) for 1 h at room temperature and then washed with TBST 3 times.

### Cell culture

Mouse aorta vascular smooth muscle cells (VSMCs) were purchased from ATCC. The cells were grown in 6-well culture plates in RPMI 1640 supplemented with 10% FBS, penicillin and streptomycin at 37 °C in a humidified atmosphere of 5% CO_2_. The medium was changed daily, and the cells were passaged after treatment with 0.05% trypsin-0.02% EDTA solution. Cells at passage 5-8 were used in the subsequent experiments. Cells were confirmed as SMCs by their typical “hill-and-valley” morphological features and by the expression of smooth muscle α-actin by immunofluorescence. Cells were made quiescent by a 48-h incubation in RPMI 1640 medium containing 0.1% FBS.

### Cytotoxicity assay

Cells were seeded in 96-well culture plates at a concentration of 5 × 10^5^ cells/ml 24 h prior to experiments. The cultured cells were stimulated with 10 μM PHPS1 or an equal volume of DMSO for 30 min and then with 100 μg/ml oxLDL for 48 h. Then, cells were washed 3 times with PBS. To evaluate the cytotoxicity of PHPS1 and DMSO, the number of cells was determined using the thiazolyl blue (MTT) test. A 10-μl volume of 5 mg/ml MTT was added to each well, and the plates were incubated for 4 h at 37 °C. The medium was removed, and the formazan crystals inside the cells were dissolved in 200 μl of DMSO. The absorbance of each well was measured at 450 nm on a microplate reader.

### BrdU incorporation assay

5-Bromo-2′-deoxyuridine (BrdU) uptake by VSMCs was measured using a kit obtained from Amersham International. Quiescent cells at 70% confluence in DMEM with 0.5% FBS were treated with different stimuli for 24 h. BrdU (1 μmol/L) was added and co-cultivated with the cells for the last 3 h of the 24-h stimulation period. BrdU incorporation into the cells was quantified using the BrdU cell proliferation assay kit (Roche Diagnostics, Indianapolis, IN, USA). The cells were counted under a microscope using a hemocytometer as previously described [20]. BrdU-labeled and unlabeled SMCs were counted in each section. The proliferation index was calculated by dividing the number of BrdU-labeled cells by the number of unlabeled cells.

### Statistics

All data are presented as the mean ± SE. Differences between groups were assessed using Student’s t-test or one-way analysis of variance for multiple comparisons. *P* values < 0.05 indicated statistical significance, and the significance levels are provided in the text.

## Results

### PHPS1 renders Ldlr^−/−^mice less susceptible to AS development

**Fig. 1:**
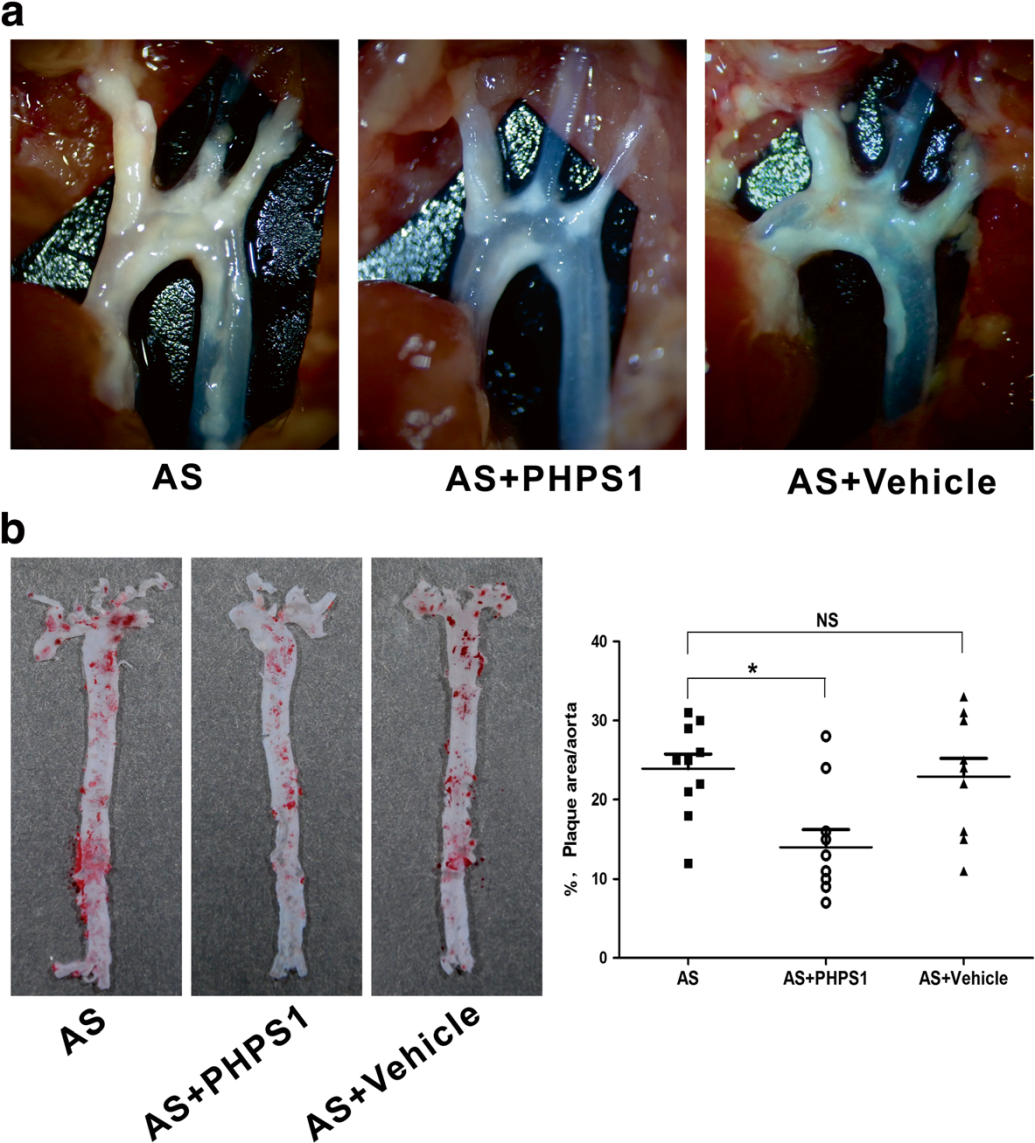
The atherosclerotic plaque area was decreased in the AS+PHPS1 group. Ldlr^−/−^ mice were fed a high-fat diet for 4 weeks. Mice in the AS+PHPS1 group were intraperitoneally injected with 3 mg/kg PHPS1 at the indicated time, and those in the AS+Vehicle group were injected with saline containing 0.5% DMSO. **a** Images of the aortic arch of Ldlr^−/−^ mice in the AS, AS+PHPS1 and AS+Vehicle groups. **b** Left: representative en face images of the Oil Red O-stained whole aorta of Ldlr^−/−^ mice. Right: quantification of the plaque area as a percentage of the aortic surface in Ldlr^−/−^ mice. Data are presented as the mean ± SE (*n* = 10 per group). *p < 0.05 vs. the AS group

**Fig. 2:**
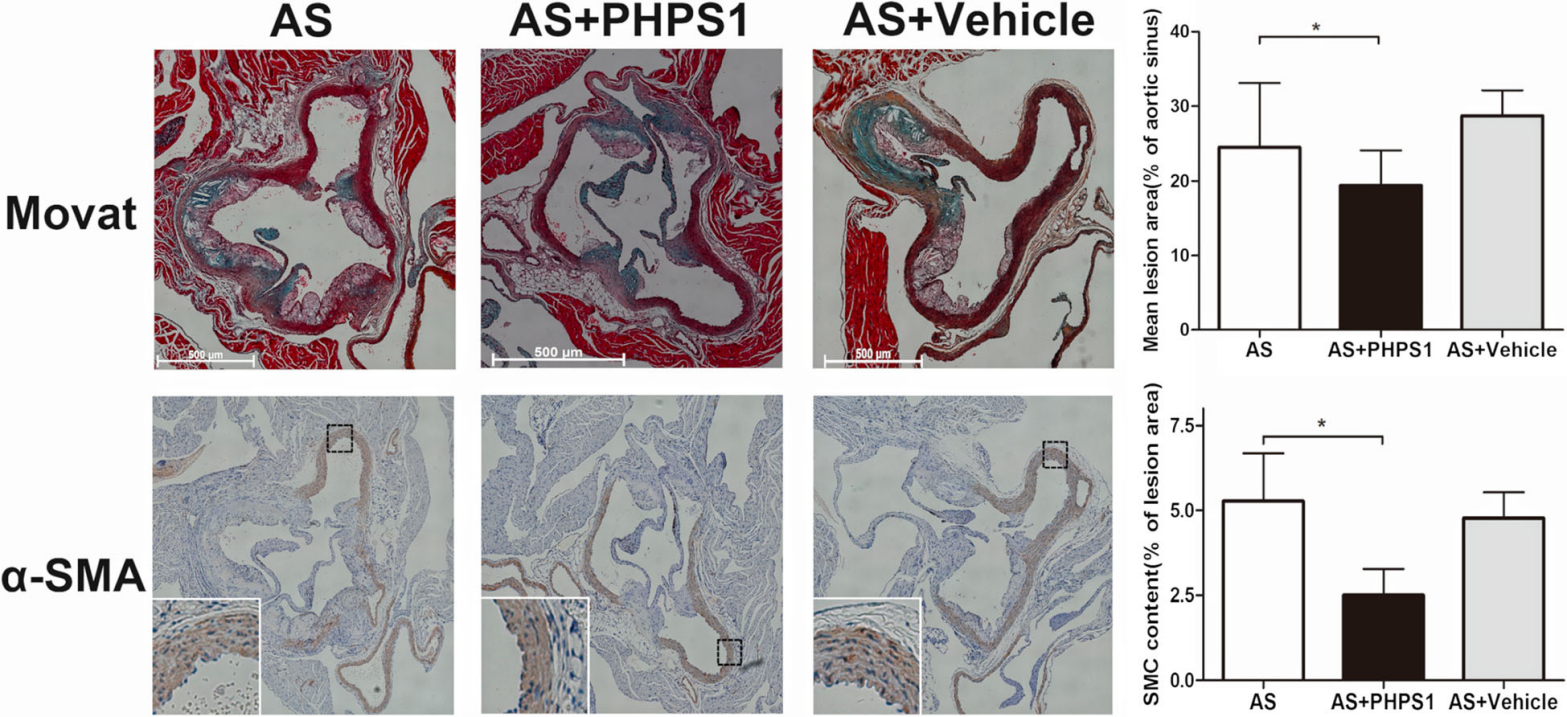
Effect of PHPS1 on atherosclerotic lesion size in Ldlr^−/−^ mice. The lesion volume/vessel volume ratio at the aortic root was analyzed by Movat staining. The number of smooth muscle cells in the plaques from the aortic root was determined by immunohistochemistry. PHPS1 treatment significantly reduced the atherosclerotic plaque area and inhibited the formation of atherosclerotic plaques. Compared with the intima in the other two groups, the intima was mildly thickened, the smooth muscle cells were arranged neatly, and smooth muscle cell proliferation was decreased in the AS+PHPS1 group. PHPS1 treatment inhibited the proliferation of vascular smooth muscle cells. Data are presented as the mean ± SE (*n* = 10 per group). *p < 0.05 vs. the AS group

To determine the pro-atherosclerotic or anti-atherosclerotic role of PHPS1, we ascertained the effect of PHPS1 on the development and progression of AS in Ldlr^−/−^ mice. No significant differences in body weight or the levels of serum glucose, TC, TG, HDL-C or LDL-C were observed among the three groups (Additional file 1: Figure S1). Remarkably, en face analysis of the aorta stained with Oil Red O revealed a significant decrease in atherosclerotic plaque size in the aorta of the AS+PHPS1 group compared with the other two groups (Fig. 1). The atherosclerotic plaque area at the aortic root was significantly decreased in Ldlr^−/−^ mice treated with PHPS1. However, Movat staining showed no significant difference between the AS group and the Vehicle group. In the AS and AS+Vehicle groups, the intimal lesion was thicker, and both the vessel and lumen were narrowed. PHPS1 inhibited neointimal formation and SMC proliferation (Fig. 2).

### PHPS1 treatment inhibits the phosphorylation of SHP2 and ERK

**Fig. 3:**
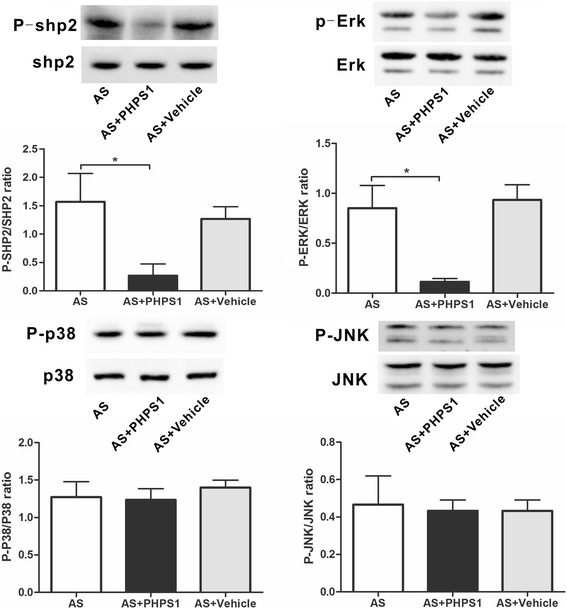
The levels of total and phosphorylated SHP2, ERK, JNK and p38 were determined by Western blot analysis. There were no statistically significant differences in the total levels of SHP2, ERK, JNK or p38^MAPK^ among the three groups (*p* > 0.05). PHPS1 suppressed the levels of phosphorylated SHP2 and ERK. Data are presented as the mean ± SE. *p < 0.05 vs. the AS group

We analyzed the total and phosphorylated levels of JNK, p38^MAPK^ and ERK1/2 by Western blot. PHPS1 suppressed SHP2 and ERK phosphorylation without affecting JNK or p38^MAPK^ activation (Fig. 3), suggesting that PHPS1 might suppress P-SHP2 and P-ERK levels and consequently inhibit the progression of early lesions in Ldlr^−/−^ mice.

### Cytotoxic effects of PHPS1 on VSMCs in vitro

**Fig. 4:**
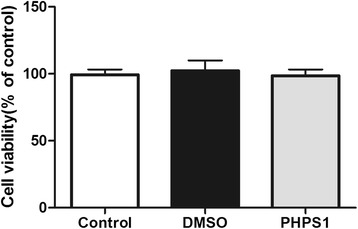
Effects of DMSO and PHPS1 on the growth and viability of VSMCs. Cultured VSMCs were stimulated with 10 μM PHPS1 or an equal volume of DMSO for 30 min and then with 100 μg/ml oxLDL for 48 h. Cell number and viability were determined by the MTT assay. Data are presented as the mean ± SE. *p < 0.05 vs. the control group

Because it was unknown whether PHPS1 is toxic to VSMCs, we treated cells with 10 μM PHPS1 or an equal volume of DMSO for 30 min. The doses of PHPS1 and DMSO used in the experiment were not toxic to the cells (Fig. 4).

### OxLDL induces ERK phosphorylation and activation and promotes VSMC proliferation

**Fig. 5:**
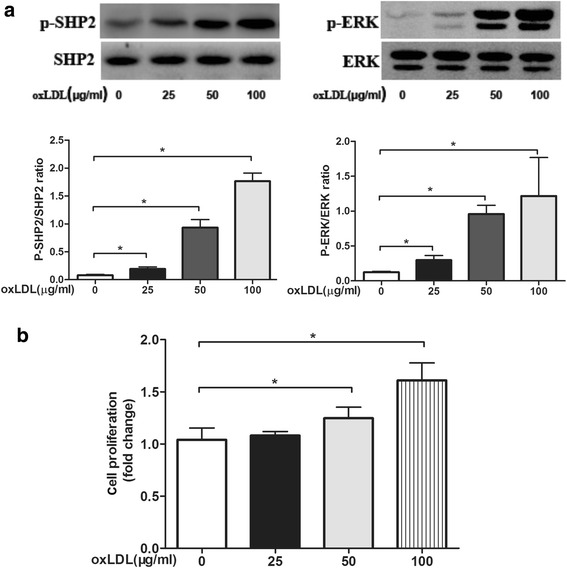
**a** Quiescent cells were treated with different concentrations (0, 25, 50 or 100 μg/ml) of oxLDL for 10 min. Treatment was terminated by washing the cells with ice-cold PBS. Cells were lysed in 100 μl of lysis buffer containing protease and phosphatase inhibitor cocktails. Protein content in the samples was determined using the bicinchoninic acid assay, and the samples were heated at 100 °C in 5× protein loading dye for 5 min. Equal amounts of protein were separated by SDS-polyacrylamide gel electrophoresis, transferred to a polyvinylidene fluoride membrane and probed with monoclonal antibodies against total and phosphorylated SHP2 and ERK1/2. OxLDL activated SHP2 in a concentration-dependent manner. Total SHP2, phosphorylated SHP2, total ERK and phosphorylated ERK levels were examined by Western blot. Relative quantification of target proteins was performed by comparing band density levels among samples. The results are reported as the mean ± SE (*n* = 3 per group). *p < 0.05 vs. the no-oxLDL treatment group. **b** Effects of SHP2 on VSMC proliferation. Smooth muscle cells were exposed to different concentrations (0, 25, 50 or 100 μg/ml) of oxLDL for 24 h. BrdU (1 μmol/L) was added, and the cells were incubated for 3 h. BrdU incorporation was quantified using the BrdU cell proliferation assay kit. The percentage of BrdU-positive VSMCs was determined. Data are presented as the mean ± SE of three independent experiments. *p < 0.05 vs. the no-oxLDL treatment group

VSMCs were incubated with 0, 25, 50 or 100 μg/ml oxLDL for 10 min. P-SHP2 and P-ERK levels were increased in the group treated with 25 μg/ml oxLDL compared with the control group and were highest in the 100 μg/ml group, revealing a concentration-dependent effect. The difference in total protein expression among groups was not statistically significant (Fig. 5a). The effect of oxLDL on VSMC proliferation was detected by the BrdU assay. As shown in Fig. 5b, stimulation with 100 μg/mL oxLDL for 24 h resulted in an approximate 50% increase in cell number, but that dose of oxLDL did not seem to affect VSMC proliferation to the same degree as 25 μg/mL oxLDL.

### PHPS1 inhibits oxLDL-induced ERK phosphorylation and VSMC proliferation

**Fig. 6:**
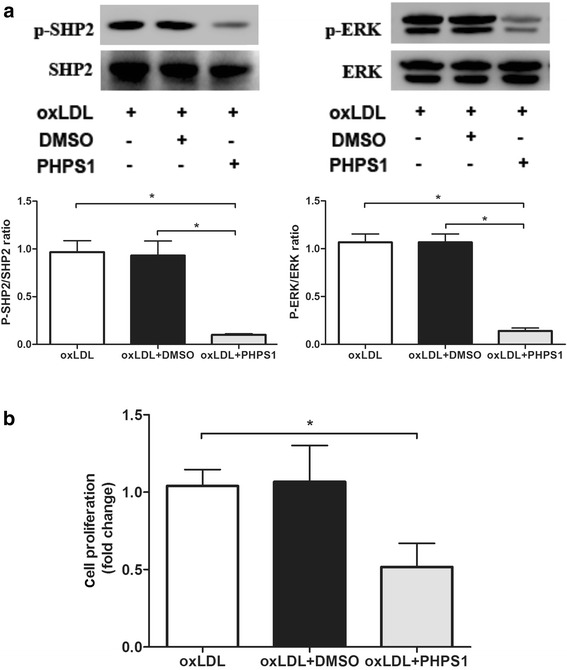
**a** Protein expression levels of total SHP2, phosphorylated SHP2, total ERK and phosphorylated ERK in VSMCs. VSMCs were treated with PHPS1 or DMSO for 30 min and then with 100 μg/ml oxLDL for 10 min. Data are presented as the mean ± SE. *p < 0.05 vs. the oxLDL group. **b** Effects of SHP2 on VSMC proliferation. After preincubation in the presence or absence of 10 μM PHPS1 or an equal volume of DMSO for 30 min at 37 °C, cells were stimulated with oxLDL (100 μg/mL) for 24 h. BrdU (1 μmol/L) was added, and the cells were incubated for 3 h. BrdU incorporation was quantified using the BrdU cell proliferation assay kit. The percentage of BrdU-positive VSMCs was determined in the three groups. Compared with the other groups, the PHPS1 treatment group showed a decrease in the percentage of BrdU-positive VSMCs. *p < 0.05 vs. the oxLDL group

Treatment of VSMCs with 100 μg/ml oxLDL increased the levels of phosphorylated SHP2 and ERK. PHPS1 attenuated P-SHP2 and P-ERK levels. In VSMCs, ERK phosphorylation was markedly inhibited after a 10-min treatment with 10 μM PHPS1. Treatment with DMSO did not affect P-SHP2 and P-ERK levels compared with control treatment (Fig. 6a). These results suggested that PHPS1 could inhibit oxLDL-induced SHP2-ERK signaling. As shown in Fig. 6b, VSMC proliferation was inhibited by PHPS1. Moreover, pretreatment with PHPS1 reversed the reinforcing effect of oxLDL on VSMC proliferation.

## Discussion

This study showed that inhibiting the tyrosine phosphatase SHP2 with PHPS1 prevented the development of AS by inhibiting VSMC proliferation. This finding may provide a better understanding of the novel biological role of the SHP2 enzyme in the pathogenesis of AS.

VSMCs participate in the development of AS [21]. Abnormal VSMC proliferation and migration contribute to atherosclerotic plaque formation, restenosis after percutaneous transluminal angioplasty and accelerated arteriopathy after cardiac transplantation [22, 23]. When chronic inflammation occurs in AS, arterial VSMCs become aberrantly regulated, leading to increased VSMC dedifferentiation and extracellular matrix formation in plaque areas [24]. Disturbances in hemodynamic forces could initiate a proinflammatory switch in the VSMC phenotype, even in the preclinical stages of AS [25]. Proinflammatory signals promote the further dedifferentiation of VSMCs in affected vessels and the propagation of pathological vascular remodeling [26, 27].

A number of studies have shown that SHP2 can promote VSMC proliferation and intimal hyperplasia [28, 29]. SHP2 was reported to be positively involved in the angiotensin II pathway, an important pathway involved in SMC proliferation [30]. SHP2 may act as an adaptor protein in the association of JAK2 with the AT_1_ receptor, thus facilitating Ang II-induced JAK2 phosphorylation and activation [31]. By enhancing the phosphorylation levels of Syk and p38^MAPK^, SHP2 facilitates the regulation of PDGF-BB-induced VSMC migration and neointima formation [32]. Induction by extracellular stimuli such as FBS, platelet-derived growth factor or insulin-like growth factor-1 enhances SHP2 levels and BrdU uptake in SMCs, suggesting that SHP2 may accelerate VSMC proliferation [28].

Our in vivo experiments showed that inhibition of SHP2 by PHPS1 had a protective effect on AS development by reducing VSMC proliferation. To evaluate the effect of SHP2 on AS, Ldlr^−/−^ mice were fed a diet containing 1.25% cholesterol for 4 weeks. As a result, early plaques formed in the aortic root. Subcutaneous injection of PHPS1 significantly inhibited VSMC proliferation and intimal thickening during the development of AS. Moreover, PHPS1 treatment significantly inhibited ERK phosphorylation in the aortic wall; ERK is a key regulator of cell proliferation [19], indicating that PHPS1 may attenuate VSMC proliferation by regulating the activation of ERK signaling cascade. Our in vitro experiment also verified the inhibitory effect of SHP2 on VSMC proliferation.

Our in vitro data showed that oxLDL increased SHP2 and ERK phosphorylation and simultaneously enhanced VSMC proliferation. However, these responses were suppressed by treatment with the SHP2 inhibitor PHPS1. These results implied that SHP2 positively regulated VSMC proliferation in response to oxLDL by regulating ERK phosphorylation. SHP2 is reported to be involved in VSMC proliferation. OxLDL promotes the VSMC phenotypic switch from the contractile phenotype to the proinflammatory phenotype, which is associated with dedifferentiation and proliferation [33, 34]. In addition, OxLDL expedites the progression of AS through the accumulation of reactive oxygen species (ROS) and activation of the MAPK stress signaling cascade and stimulates the expression and secretion of ET-1 [35]. However, the relationship between SHP2 and oxLDL in VSMC proliferation remains unknown. This study showed that oxLDL stimulation further increased SHP2 phosphorylation, SHP2 activation by ERK phosphorylation promoted VSMC proliferation, and pretreatment with the SHP2 inhibitor PHPS1 inhibited the oxLDL-induced activation of SHP2 and VSMC proliferation. These results also indicated that SHP2 promoted VSMC proliferation via activation of the ERK signaling cascade.

## Conclusion

The results of the present study suggest that the SHP2 inhibitor PHPS1 can inhibit VSMC proliferation by reducing the phosphorylation of ERK and suppressing activation of signaling cascade. PHPS1 is beneficial for delaying the development of AS, and it is a potential therapeutic target for cardiovascular diseases associated with vascular remodeling.

## Additional file

Additional file 1: **Figure S1.** Ldlr^−/−^ mice fed with a diet containing 10% fat, 10% yolk powder and 1.25% cholesterol for 4 weeks were administered PHPS1 or vehicle at a dose of 3 mg/kg every day during the fourth week. Body weight (A); serum glucose levels (B); and TC, TG, LDL-C and HDL-C levels (C) were measured. Data are reported as the mean ± SE (*n* = 10 per group). *p < 0.05 vs. the AS group. (TIF 20388 kb)

## Abbreviations

AS: Atherosclerosis
BrdU: 5-bromo-2′-deoxyuridine
HDL-C: High-density lipoprotein cholesterol
LDL-C: Low-density lipoprotein cholesterol
OCT: Optimal cutting temperature
PBS: Phosphate-buffered saline
PHPS1: Phenylhydrazono pyrazolone sulfonate 1
PTK: Protein tyrosine kinase
PTP: Protein tyrosine phosphatase
PTPN11: Protein tyrosine phosphatase N 11
SHP2: Src homology 2 domain-containing protein tyrosine phosphatase 2
TC: Total cholesterol
TG: Triglyceride
VSMCs: Vascular smooth muscle cells
