# Supplementary figures and images for: SHP2 inhibitor PHPS1 protects against atherosclerosis by inhibiting smooth muscle cell proliferation
Source: BMC Cardiovasc Disord. 2018 Apr 27;18:72. doi: 10.1186/s12872-018-0816-2 (PMC5923012; doi:10.1186/s12872-018-0816-2)

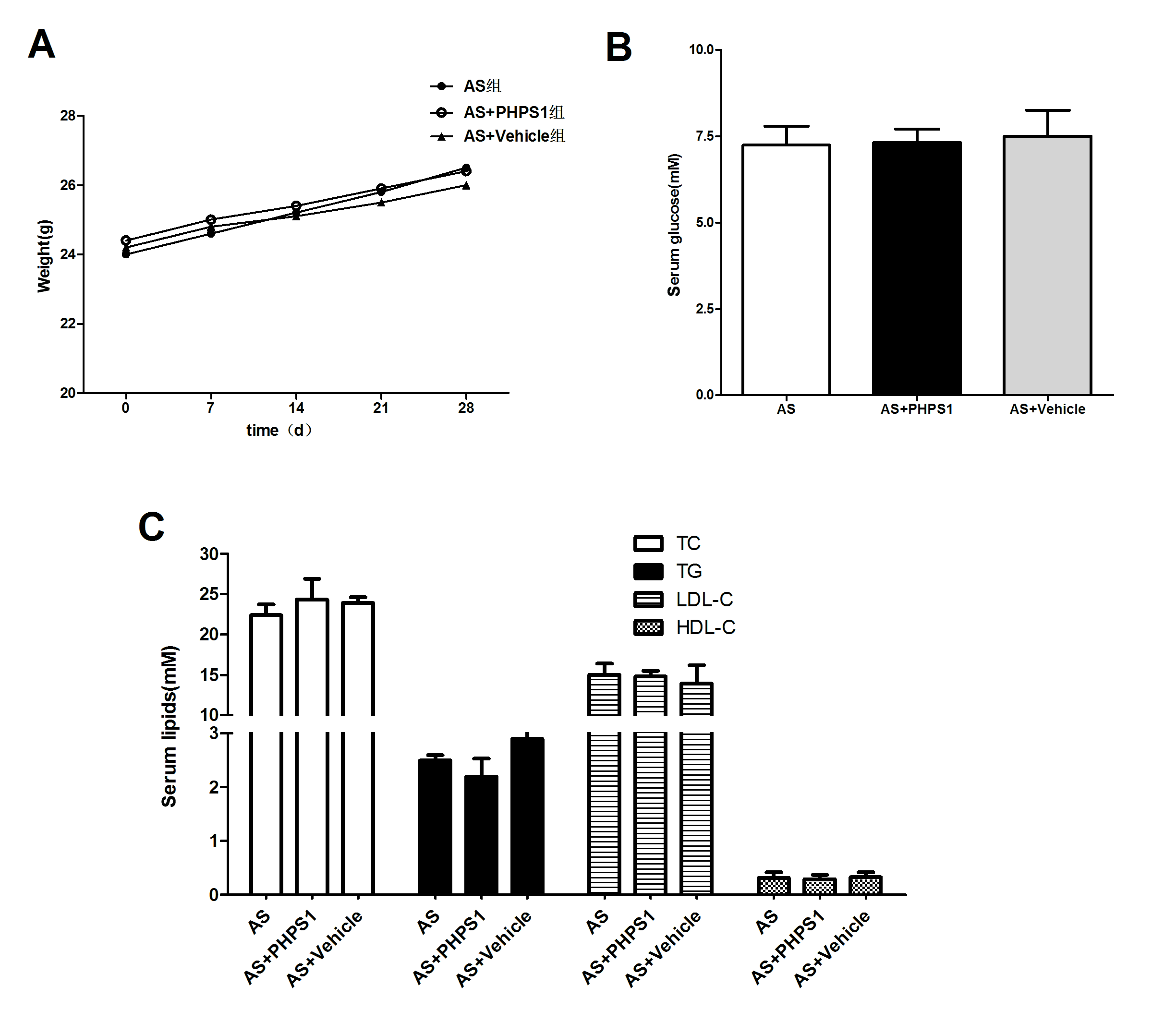

Supplement: Supplementary file 1 — Figure S1. Ldlr−/− mice fed with a diet containing 10% fat, 10% yolk powder and 1.25% cholesterol for 4 weeks were administered PHPS1 or vehicle at a dose of 3 mg/kg every day during the fourth week. Body weight (A); serum glucose levels (B); and TC, TG, LDL-C and HDL-C levels (C) were measured. Data are reported as the mean ± SE (n = 10 per group). *p < 0.05 vs. the AS group. (TIF 20388 kb) [file 12872_2018_816_MOESM1_ESM.tif]
